# Impact of comorbidity on lung cancer mortality - a report from the Liverpool Lung Project

**DOI:** 10.3892/ol.2015.2916

**Published:** 2015-01-28

**Authors:** MICHAEL W MARCUS, YING CHEN, STEPHEN W DUFFY, JOHN K FIELD

**Affiliations:** 1 Roy Castle Lung Cancer Research Programme, Department of Molecular and Clinical Cancer Medicine, Liverpool Cancer Research UK Centre, Institute of Translational Medicine, The University of Liverpool, Liverpool L3 9TA, UK; 2 Wolfson Institute of Preventive Medicine, Barts and the London School of Medicine and Dentistry, Queen Mary University of London, Charterhouse Square, London EC1M 6BQ, UK

**Keywords:** comorbidities, other-cause mortality, lung cancer-specific mortality

## Abstract

The aim of the present study was to apply the Charlson comorbidity index (CCI) to evaluate the impact of comorbidity on lung cancer mortality in individuals not exhibiting lung cancer at the commencement of follow-up. Data from 9,579 participants without lung cancer were extracted from the Liverpool Lung Project between 1999 and 2010 and linked to the Hospital Episode Statistics database. The occurrence of comorbidities was assessed one year prior to the individual inclusion date. CCI scores were computed and Cox regression analysis was used to evaluate the association between comorbidity and lung cancer mortality using a competitive risk approach. During a median follow-up of 11 years, 1,320/9,579 (13.7%) individuals developed lung cancer, of which 886 (67.1%) succumbed to lung cancer and 875 of the 9,579 individuals (9.1%) succumbed due to other causes. The severity of comorbidity was associated with higher lung cancer-specific mortality; low to moderate comorbidity exhibited a hazard ratio (HR) of 2.86 [95% confidence interval (CI), 1.17–7.02] and severe comorbidity exhibited an HR of 5.16 (95% CI, 2.07–12.89). Furthermore, the CCI score determined that the severity of comorbidity increased the risk of lung cancer-specific mortality. Thus, CCI score is a good predictor of lung cancer-specific mortality and the use of comorbidity burdens in the clinical management of lung cancer is recommended.

## Introduction

Lung cancer is the leading cause of cancer-related mortality in the majority of developed countries, with the mortality rate exceeding that of colon, breast and prostate cancer combined (1,2). Lung cancer is predominantly a disease of the elderly, with an average age at diagnosis of ~60–70 years, and often presents late at an advanced disease stage (3,4). Due to increasing longevity and rapid ageing populations, the number of individuals with more than one comorbid condition is expected to increase sharply in the forthcoming decades (5,6). This increase may result in an increase in the incidence of lung cancer and the comorbidity burden may lead to increased overall and/or lung cancer-specific mortality.

A number of indices for classifying the burden of illness in patients have been developed (7), many of which have been investigated for use in oncology settings (8–10). The Charlson comorbidity index (CCI) is the most widely used indices in prognostic medicine (11); it is a simple, readily applicable and valid weighted index developed for estimating the risk of mortality from comorbid diseases in longitudinal studies. The CCI was developed using the ninth revision of the International Classification of Diseases (ICD)-9 diagnosis codes, however, it has yet to be validated using ICD-10 (12,13).

Previous studies have determined that the CCI is a good prognostic tool for various outcomes, including mortality (14–17). Certain studies have evaluated its impact in predicting the effect of comorbidity on lung cancer-specific mortality (18–20); however, to the best of our knowledge no previous study has evaluated the impact of comorbidity and mortality in individuals without lung cancer at the commencement of follow-up. Therefore, the aim of the present study was to apply the CCI to evaluate the impact of comorbidity on lung cancer-specific mortality in a prospective cohort study.

## Patients and methods

### Study design and participants

The present study is a based on the analysis of prospectively collected data from 9,579 participants of the Liverpool Lung Project (LLP), aged 45–79 years, who did not exhibit lung cancer at baseline (at the commencement of follow-up). Cohort entry into the LPP commenced on January 1, 1999 and each individual was followed from cohort entry until mortality, or to the end of the follow-up period on December 31, 2010, whichever occurred first. Details of the study design and data collection methods of the LLP has been reported previously (21). Briefly, the LLP is a population-based case control and cohort study of the early detection of lung cancer in Liverpool, United Kingdom. Study subjects (n=326,000) were randomly selected from all residents within the designated Liverpool postcode study area via the list of general practitioners held by each National Health Service (NHS) Primary Care Trust. Following receipt of informed consent, a standardised lifestyle questionnaire was conducted to obtain detailed information regarding the socioeconomic and demographic characteristics of the subjects. The LLP project was approved by the Liverpool Research Ethic committee.

### Data sources

Clinical and epidemiological data from the LLP was linked to the Hospital Episode Statistics database (HES). The HES database is a population-based administrative database that records details of all admissions, outpatient appointments, and accidents and emergencies attendances at National Health Service hospitals in England. HES was originally conceived in 1987 and currently processes >125 million admitted patient, outpatient, and accident and emergency records each year (22). HES data consists of primary and secondary diagnoses coded according to ICD-10, procedures, as well as hospital admission and discharge dates. Lung cancer incidence and mortality status was recorded annually for each participant in the present study via the Office for National Statistics (ONS), the North West Cancer Intelligence Service or hospital case notes, and linked to the abovementioned databases.

### Classification of comorbidity

Comorbidities were defined as the date of hospitalisation (based on ICD-10 diagnosis codes) as recorded in the HES database. The CCI was derived from the secondary diagnosis codes that used the number and severity of comorbid disease to compute the CCI score (CCIS). CCIS is based on 19 chronic conditions each assigned with a weight. The assigned weight for each chronic condition was as follows: Weight 1, myocardial infarction, congestive heart failure, peripheral vascular disease, cerebrovascular disease, dementia, chronic pulmonary disease, connective tissue disease, peptic ulcer disease, mild liver disease and diabetes; weight 2, hemiplegia, moderate or severe renal disease, diabetes with end organ damage, any tumour excluding lung cancer, leukaemia and lymphoma; weight 3, moderate or severe liver disease; and weight 6, metastatic solid tumour or AIDS (11). The occurrence of comorbidities was assessed from one year prior to the date on which subjects were enrolled in the LLP. CCIS was calculated as the sum of the weighted scores of all the comorbid conditions and divided into three groups: CCIS, 0=no comorbidity; CCIS, 1–2=low to moderate comorbidity; and CCIS, ≥3=severe comorbidity. A CCIS of 0 was used as the reference for all analyses.

### Classification of mortality

The mortality outcome was defined as the primary cause of mortality recorded by the ONS, the North West Cancer Intelligence Service or hospital case notes. The causes of mortality were classified into the following two categories using the ICD-10 codes: i) Mortality caused by primary lung cancer and ii) mortality due to other causes. Mortality from other causes was defined as any mortality excluding mortality from lung cancer, and lung cancer-specific mortality was defined as mortality that resulted from any of the topographic subcategories of code C34 according to ICD-10.

### Statistical analysis

Descriptive statistics are used to present the frequency distribution of the baseline study characteristics as numbers and percentages or as the mean ± standard deviation. The effect of baseline characteristics on mortality status was evaluated at the univariate level by performing a log-rank test for the categorical variables and univariate Cox proportional hazard regression for the continuous variables. All of the baseline characteristics that were statistically significant (P<0.05) in the univariate analyses were included in the multivariate Cox model. The aim of the present study was to estimate the effect of comorbidity on lung cancer-specific mortality; however, competitive risks often arise when the occurrence of one type of event prevents the occurrence of other types of events (23). In the current case, other events, such as mortality from other causes may compete with lung cancer-specific mortality and, therefore, bias the estimated effects (24). In the presence of competing risks, the standard Cox proportional hazards model is inadequate as the cause-specific Cox model treats mortality from other causes as a censored observation (25); therefore, mulitivariate Cox regression models were used in the present study to compute hazard ratios (HRs) and the corresponding confidence intervals (CIs) to determine the association between comorbidity and mortality status using a competitive risk approach (26). All analyses were performed using SAS^®^ statistical software (version 9.3; SAS Institute, Inc., Cary, NC, USA) and Stata software (version 13.1; StataCorp, College Station, TX, USA). All reported P-values were two-sided and P<0.05 was considered to indicate a statistically significant difference.

## Results

A total of 9,579 participants without lung cancer were extracted from the LLP between 1999 and 2010, and linked to the HES database. During a median follow-up period of 11 years, 1,320/9,579 participants (13.8%) developed lung cancer, of which 886 (67.1%) succumbed to lung cancer and 875 of the 9,579 individuals (9.1%) succumbed due to other causes. Table I indicates the distribution of the baseline study-specific risk factors by mortality status, with emphasis on the comparison between lung cancer mortality and mortality from other causes. The majority of mortalities were recorded in male participants, with more males succumbing due to other causes. Of the participants that succumbed to lung cancer, a higher proportion smoked for a longer period of time compared with their counterparts that succumbed due to other causes. Additionally, significant differences were noted in other risk factors between the lung cancer-specific and other-cause mortality groups, including education (P<0.0001), marital status (P=0.003), family history of lung cancer (P=0.021) and CCIS (P<0.001).

Table II presents the frequencies of comorbidities identified using CCI for lung cancer-specific and other-cause mortality. The most common comorbidities among those individuals who succumbed to lung cancer were chronic pulmonary disease (30.9%), metastatic solid tumour (24.8%), diabetes without complications (10.3%) and peripheral vascular disease (8.7%). Among those that succumbed due to other causes, the most common comorbidities were chronic pulmonary disease (40.5%), lymphoma/leukaemia (38.6%) metastatic solid tumour (22.7%) and diabetes without complications (17.5%). The final multivariate cox regression model is presented in Table III. Using no comorbidity as the reference, low to moderate comorbidity (HR, 2.86; 95% CI, 1.17–7.02) and severe comorbidity (HR 5.16; 95% CI, 2.07–12.89) were significantly associated with lung cancer-specific mortality following adjustment for age, gender, smoking duration, education and marital status.

## Discussion

Despite increased recognition of the importance of comorbidities in the long-term prognosis of lung cancer, evidence in the literature of the causal link between comorbidities and the risk of lung cancer-specific mortality remains scarce. In the present study, CCIS was used to identify that the severity of comorbidities increases the risk of lung cancer-specific mortality.

To the best of our knowledge, the association between comorbidities and an increased risk of lung cancer-specific mortality identified in the present study is unique; the CCI index was prospectively applied to evaluate the impact of comorbidities on lung cancer-specific mortality in a cohort of participants not exhibiting lung cancer at baseline, using a competitive risk approach. Mortality from other causes may be a complicating factor from a pathophysiological point of view; however, for patients and clinicians, the most important question is the probability of lung cancer mortality occurring. The present study adequately answered this question and demonstrated that the severity of comorbidities increases the probability of lung cancer mortality. Furthermore, a dose-response association was observed between the severity of comorbidity and lung cancer-specific mortality, taking into account of mortality due to other causes.

Previous studies have reported an association between severe comorbidity and lung cancer-specific mortality. For example Jørgensen *et al* (27) reported an HR of 1.29 (95% CI, 1.03–1.60) in a large population-based case-control study that used the CCI to quantify comorbidity burden in elderly cancer patients. Additionally, a large randomised trial using the CCI reported an association between any comorbidity versus no comorbidity and poor survival of lung cancer patients [HR, 1.28 (95% CI, 1.09–1.5)] (28). The frequency and severity of comorbidity have also been reported as a more useful predictor of survival for lung cancer patients who underwent surgery compared with the analysis of an individual comorbid condition (29). The result of the present study is not comparable to the aforementioned studies, as these studies evaluated the association between comorbidity and mortality in patients with lung cancer, whereas the association reported in the present study is between comorbidity and mortality in individuals not exhibiting lung cancer at the commencement of follow-up.

The strengths of the present study include the population-based design, the large sample size, the long follow-up period and the use of ONS data to minimise the risk of unavailable mortality information. In addition, detailed information concerning the potential risk factors in the LLP were collected using standardised questionnaires. However, the result of the present study must be considered in the light of a number of limitations. First, the coding of the HES data is primarily for administrative purposes and, thus, susceptible to coding bias as the coding guidelines only require the recording of comorbidities that are considered relevant to hospital admissions, possibly leading to the underreporting of comorbidities in patients with serious acute conditions (30). In addition, coding is not standardised between the various NHS trusts, which may have resulted in misclassification and, therefore, underestimation or overestimation of the diagnosis of comorbidities (30). Secondly, the classification of total severity in multiple comorbid conditions may have a multiplicative rather than additive effect. Classifying comorbidity burden as a discrete or dichotomous variable, a sum of scores or as the most severe condition present may result in an underestimation of the burden of multiple diseases on prognosis. However, previous studies are in support of the approach of combining individual comorbidity conditions utilised in the present study (31).

Lung cancer-specific mortality may be attributed to higher comorbidity burden caused by other diseases associated with smoking, such as cardiovascular diseases and chronic pulmonary diseases (32), which may cofound the association determined in the present study. However, the significant number of smokers investigated by univariate analysis supports the established evidence that lung cancer-specific mortality is associated with smoking (Table I). In addition, the present data was adjusted for smoking history in the multivariate model. Furthermore, a high lung cancer-specific mortality was observed in the present study population, which is a reflection of the overall trend in lung cancer incidence and mortality in the Liverpool region of the UK. Liverpool has the highest incidence (88.9/100,000 individuals) and mortality rate (79.7/100,000 individuals) of lung cancer compared with the incidence (48.0/100,000 individuals) and mortality rate (39.7/100,000 individuals) in England as a whole (National Cancer Intelligence Network, 2012) (33).

In conclusion, to the best of our knowledge, the present study was the first to document the impact of comorbidities on lung cancer-specific mortality in the UK using comorbidity information from the HES database in a randomly selected population cohort of 9,579 individuals. The CCI was a good predictor of lung cancer-specific mortality, in agreement with a previously conducted meta-analysis (28). Therefore, there is potential to utilise HES data for prognostic purposes, which may contribute to the improved clinical management of lung cancer patients in the future.

## Figures and Tables

**Table I:** Characteristics of the study population (n=9579) by mortality status.

Characteristic	Alive (n=7818; 81.6%)	Lung cancer mortality (n=886; 9.3%)	Other-cause mortality (n=875; 9.1%)	P-value
Age, years (mean ± SD)^a^	63.1±8.3	67.9±7.5	69.6±7.3	<0.0001
Gender^b^, n (%)				<0.0001
Male	3637 (77.4)	509 (10.8)	556 (11.8)	
Female	4181 (85.7)	377 (7.7)	319 (6.5)	
Smoking duration^a^				<0.0001
Mean ± SD	16.6±18.2	41.1±14.4	26.5±19.6	
Median	10	44	29	
BMI^a^, mean ± SD	27.0±4.8	27.3±11.1	26.8±5.3	0.0001
Education^b^, n (%)				<0.0001
≤High school graduate	1235 (93.1)	29 (2.2)	62 (4.7)	
≥College	6583 (79.8)	857 (10.4)	813 (9.9)	
Marital status^b^, n (%)				<0.0001
Married	4903 (83.5)	505 (8.6)	462 (7.9)	
Other	2277 (83.9)	183 (6.7)	255 (9.4)	
Family history of lung cancer^b^, n (%)				0.0030
No	6309 (80.9)	751 (9.6)	739 (9.5)	
Early onset (≤60 years)	458 (86.1)	39 (7.3)	35 (6.6)	
Late onset (≥60 years)	1051 (84.2)	96 (7.7)	101 (8.1)	
CCIS^b^, n				<0.0001
0	423 (98.6)	5 (1.2)	1 (0.2)	
1–2	4027 (92.8)	229 (5.3)	84 (1.9)	
≥3	3368 (70.0)	652 (13.6)	790 (16.4)	

a P-values derived from univariate Cox regression;

b P-values derived from the log-rank test.

SD, standard deviation; BMI, body mass index; CCIS, Charlson comorbidity index score.

**Table II:** Comorbidities identified using the Charlson comorbidity index for lung cancer-specific and other-cause mortality.

Comorbidity	Lung cancer mortality, n (%) (n=886)	Other-cause mortality, n (%) (n=875)	P-value^a^
Acute myocardial infarct	39 (4.4)	139 (15.9)	0.01
Congestive heart failure	19 (2.1)	149 (17.0)	0.0003
Peripheral vascular disease	77 (8.7)	127 (14.5)	<0.0001
Cerebrovascular disease	30 (3.4)	102 (11.5)	0.13
Dementia	1 (0.1)	28 (3.2)	0.05
Chronic pulmonary disease	274 (30.9)	354 (40.5)	<0.0001
Rheumatic disease	23 (2.6)	36 (4.1)	0.55
Peptic ulcer	17 (1.9)	43 (4.9)	0.88
Mild liver disease	13 (1.5)	41 (4.7)	0.40
Diabetes without complications	91 (10.3)	153 (17.5)	0.54
Diabetes with complications	7 (0.8)	20 (2.3)	0.15
Hemiplegia/paraplegia	10 (1.1)	30 (3.4)	0.59
Renal disease	11 (1.2)	106 (12.1)	0.04
Lymphoma/leukaemia	57 (6.4)	338 (38.6)	0.002
Moderate or severe liver disease	4 (0.5)	12 (1.4)	0.23
Metastatic solid tumour	220 (24.8)	199 (22.7)	<0.0001
lymphoma	3 (0.3)	24 (2.7)	0.25
Leukaemia	2 (0.2)	8 (0.9)	0.69
Autoimmune deficiency syndrome	-	-	-

a P-values derived from the log-rank test.

**Table III:** Multivariate analysis to determine the association between comorbidity and lung cancer-specific mortality (adjusted for age, gender, smoking duration, education and marital status).

Covariate	Hazard ratio (95% CI)	P-value
CCIS		
0	Reference	
1–2	2.86 (1.17–7.02)	0.002
≥3+	5.16 (2.07–12.89)	0.0004
Age	1.01 (1.00–1.03)	<0.0001
Gender (male vs. female)	1.34 (1.17–1.53)	<0.0001
Smoking duration (years)	1.03 (1.03–1.04)	<0.0001
Education (high vs. low)	0.28 (0.19–0.41)	<0.0001
Marital status (married vs. other)	0.84 (0.73–0.96)	0.009

CCIS, Charlson comorbidity index score; CI, confidence interval.
